# Identification and functional validation of *ACSL1* as a biomarker regulating ferroptosis in nucleus pulposus cell

**DOI:** 10.1042/BSR20241414

**Published:** 2025-04-02

**Authors:** Yichi Zhou, Ke Wang, Min Ren, Jiebin Wang, Fanglin Wang, Bingbing Zhuang, Lin Chen, Zhiqiang Zhang, Changsheng Wang

**Affiliations:** 1 Department of Spine Surgery, The First Affiliated Hospital of Fujian Medical University, 20 Cha Zhong Road, Taijiang District, Fuzhou, Fujian Province, 350000, China; 2 Spine Center, Wuhan Fourth Hospital, 473 Hanzheng Road, Qiaokou District, Wuhan, Hubei Province, 430000, China; 3 Fujian Key Laboratory of Natural Medicine Pharmacology, School of Pharmacy, Fujian Medical University, Fuzhou, Fujian, China

**Keywords:** ACSL1, ferroptosis, intervertebral disc degeneration (IVDD), intervertebral disc microenvironment, nucleus pulposus cell

## Abstract

Intervertebral disc degeneration (IVDD) is a prevalent musculoskeletal disorder characterized by the deterioration of nucleus pulposus (NP) cells, leading to significant impairments in patients’ quality of life. Elucidating the molecular mechanisms underlying IVDD is essential for developing effective therapeutic strategies. In this study, we utilized weighted gene co-expression network analysis to identify key module eigengenes (MEs) from the GSE124272 dataset, combined with differential gene expression analysis to pinpoint differentially expressed genes (DEGs). Functional enrichment analysis revealed that MEs were primarily associated with lipid metabolism and immune response, while DEGs were enriched in immune response and cell proliferation pathways. By integrating MEs, DEGs, and ferroptosis-related genes, we identified six hub genes (acyl-CoA synthetase long-chain family member 1 [*ACSL1*], *BACH1*, *CBS*, *CP*, *AKR1C1*, and *AKR1C3*). Consensus clustering analysis classified samples into two immune-related subgroups, C1 and C2, with single-sample gene set enrichment analysis demonstrating distinct immune scores between the subgroups. Notably, *ACSL1* showed the strongest correlation with immune cell infiltration and was significantly up-regulated in the C1 subgroup, which exhibited higher immune scores. *In vitro* experiments confirmed elevated *ACSL1* expression in an IL-1β-induced degenerative NP cell model. Silencing *ACSL1* improved cell viability, reduced apoptosis, and restored mitochondrial membrane potential, alongside significant changes in intracellular Fe2+, malondialdehyde, and glutathione levels. *In vivo* experiments further validated increased *ACSL1* expression in intervertebral disc tissues of IVDD rats. Collectively, these findings highlight *ACSL1* as a potential biomarker for the early diagnosis of IVDD and a promising therapeutic target.

## Introduction

Intervertebral disc degeneration (IVDD) is a primary cause of lower back pain [1]. As degeneration progresses, IVDD can lead to severe symptoms such as neuropathic pain, muscle weakness, and spinal deformity, imposing substantial economic burdens on individuals and healthcare systems [2]. While aging, mechanical stress, and local inflammation are commonly believed to contribute to IVDD onset [3], the biological mechanisms underlying IVDD progression remain incompletely understood. Current treatments for IVDD primarily focus on alleviating symptom and surgical interventions, yet traditional therapeutic approaches often fall short in promoting cellular-level disc regeneration. Therefore, in-depth research and exploration into IVDD are essential for developing more therapeutic strategies.

Ferroptosis is a form of programmed cell death characterized by cellular iron overload and lipid peroxidation [4]. Excessive iron can lead to the accumulation of reactive oxygen species (ROS) via the Fenton reaction [5]. Subsequently, the excess ROS disrupt mitochondrial function, leading to oxidative stress, lipid peroxidation, and ultimately cell death [6]. Presently, extensive research has demonstrated widespread ferroptosis in degenerative nucleus pulposus (NP) cells [7]. Additionally, as disc degeneration progresses, microvessels penetrate the disc through ruptured cartilage endplates, facilitating the ingress of immune cells [8]. Remarkably, recent research by Liu et al. [9] suggests that ferroptosis plays a role in promoting IVDD progression by triggering immune cell infiltration, further exacerbating disc degeneration within this altered microenvironment.

Acyl-CoA synthetase long-chain family member 1 (*ACSL1*) is known to exert significant influence on fatty acid metabolism and has been implicated in ferroptosis across various diseases [10–12]. However, the specific mechanism underlying *ACSL1*’s regulation of ferroptosis in degenerative NP cells remains elusive. In this study, we conducted a comprehensive bioinformatics analysis to identify key hub genes associated with IVDD, with *ACSL1* emerging as a significant candidate. We further examined the immune profile of degenerative intervertebral discs, identifying *ACSL1* as a potential biomarker for IVDD through correlation analysis. *In vitro* and *in vivo* experiments confirmed *ACSL1*’s critical role in the early stages of IVDD progression. Our findings suggest that *ACSL1* may serve as a biomarker for the early diagnosis of IVDD, emphasizing the need for further research into its diagnostic potential.

## Materials and methods

### Antibodies and reagents

For western blot analysis, the following primary antibodies were used: ACSL1, GPX4, SLC7A11, and β-actin. Anti-HRP was utilized as the secondary antibody. IL-1β (HY-P73149), erastin (HY-15763), and ferrostatin-1 (HY-100579) were purchased from MCE.

### Data collection and study design

**Figure 1:**
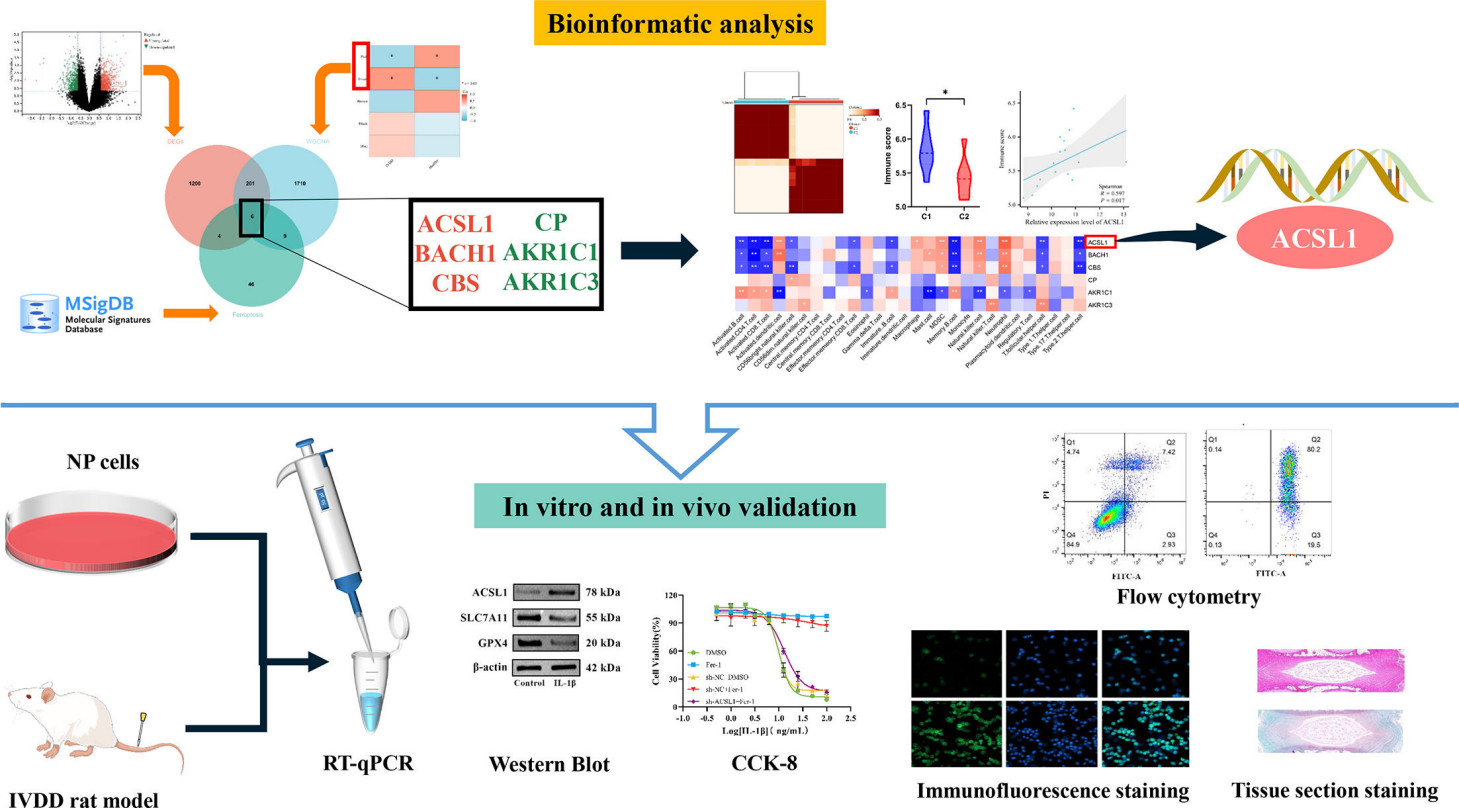
Diagram describing the research methods used in the study.

The RNA sequencing data and clinical data were obtained from the GSE124272 [13] dataset of Gene Expression Omnibus (GEO) database, encompassing peripheral blood samples from eight healthy volunteers and eight individuals diagnosed with IVDD. The datasets GSE23130, GSE153761, and GSE167199 were used as validation cohorts to verify *ACSL1* expression. Sixty-five genes associated with ferroptosis were extracted from MSigDB ( Table S1), while immune-related gene sets used for consensus clustering and immune scoring were acquired from ImmPort database (Table S2). The overall research design, including the experimental procedures and analysis workflow, is illustrated in Figure 1.

### Weighted gene co-expression network analysis

We calculated the median absolute deviation (MAD) for each gene based on gene expression profiles and removed the bottom 50% of genes with the smallest MAD values. Subsequently, we utilized the ‘goodSamplesGenes’ method from the R software package WGCNA to eliminate outlier genes and samples [14]. Furthermore, we constructed a scale-free co-expression network using weighted gene co-expression network analysis (WGCNA), employing β as a soft-thresholding parameter to emphasize strong correlations between genes while penalizing weak correlations. After determining a power of 9, the adjacency matrix was transformed into a topological overlap matrix (TOM) to assess the network connectivity of genes, with the corresponding dissimilarity calculated as (1-TOM) [15]. We applied the Benjamini–Hochberg procedure to adjust for multiple testing and control the false discovery rate (FDR) when identifying significant correlations between gene modules and clinical traits. Additionally, we utilized the R package ggplot2 to generate a heatmap depicting the correlation between WGCNA modules and clinical features.

### Differential gene expression analysis and functional enrichment analysis

We conducted differential gene expression analysis on the GSE124272 dataset using the R package limma [16]. The lmFit function was utilized to perform multiple linear regression on the expression profile dataset. Subsequently, the eBayes function was employed to compute moderated t-statistics, moderated F-statistics, and log-odds of differential expression through empirical Bayes moderation of standard errors toward a common value. Differentially expressed genes (DEGs) were determined based on a fold-change threshold of 1.5 and a significance level of 0.05.

To explore the potential characteristics of key module eigengenes (MEs) identified by WGCNA and DEGs, we performed Gene Ontology (GO) and Kyoto Encyclopedia of Genes and Genomes (KEGG) enrichment analyses using the R package clusterProfiler. The minimum gene set was set to 5, and the maximum gene set was set to 5000. A *P* value less than 0.05 and an FDR less than 0.25 were considered statistically significant. Additionally, we further annotated the functional aspects of these datasets using the online tool Metascape [17], respectively.

### Immune cell infiltration analysis, consensus clustering, and immune scoring

We utilized the single sample gene set enrichment analysis (ssGSEA) method [18] from the R package GSVA to evaluate the infiltration levels of 28 immune cell types in IVDD, followed by visualization using the R package ggplot2. Subsequently, the R package ConsensusClusterPlus [19] was employed for sample consensus clustering, categorizing the samples into distinct immune subtypes. Furthermore, we utilized immune gene sets from the ImmPort database to evaluate the immune scores of samples belonging to different subtypes, aiming to assess the immune profiles of distinct immune subtypes.

### Cell culture and treatment

Primary human NP cells were purchased from iCell (Cellverse Bioscience, China) and cultured in DMEM/F12 medium (Invitrogen, U.S.A.) supplemented with 10% fetal bovine serum (Invitrogen, U.S.A.) and 1% penicillin–streptomycin at 37°C in a humidified atmosphere containing 5% CO_2_. The medium was changed twice a week, and experiments were conducted after the cells reached the second passage.

To induce IVDD in NP cells, we subjected them to 10 ng/ml IL-1β treatment and incubated them for seven days. To validate the link between ferroptosis in NP cells and IL-1β treatment, we supplemented the cells with ferrostatin-1 (Fer-1, 1 μM) or erastin (5 μM).

### Intracellular Fe^2+^, MDA, and GSH detection

To assess intracellular iron levels, cells were harvested, lysed, and then subjected to an iron assay according to kit instructions (HRK0522, Herui biotechnologies, China). The optical density value was measured at 520 nm using a microplate reader (Thermo Fisher Scientific, U.S.A.).

The malondialdehyde (MDA) content assay kit (A003-4-1, Jiancheng Biotechnology, China) was employed to measure intracellular MDA levels using the thiobarbituric acid (TBA) reactive assay. Cells were collected and processed according to the kit instructions to determine the MDA content as previous described [20]. Briefly, MDA reacts with TBA under acidic conditions at 90–100°C, producing a pink MDA-TBA conjugate. The absorbance of the conjugate was measured at 532 nm using a microplate reader (Thermo Fisher Scientific, U.S.A.).

Glutathione (GSH) content was determined using a GSH assay kit (HRK0310; Herui biotechnologies, China) according to the manufacturer’s instructions. The GSH concentration was quantified by measuring the absorbance at 412 nm using a microplate reader (Thermo Fisher Scientific, U.S.A.). All assays were performed in triplicate.

### Short hairpin RNA transfection

We utilized plasmids containing short hairpin RNAs (shRNAs) targeting *ACSL1* and negative control shRNAs (sh-NC) plasmids (all from GenePhama, Shanghai, China). Plasmids were transfected into NP cells using Lipofectamine™ 3000 transfection reagent (Invitrogen, U.S.A.) according to the manufacturer’s instructions.

### CCK-8 assays

Cell Counting Kit-8 (C0037, Beyotime Biotechnology, China) was used to assess cell viability following the manufacturer’s instructions. NP cells with different treatment were seeded onto 96-well plates at a density of 1500 cells per well and cultured for 24 h at 37°C in a 5% CO_2_ incubator. Next, 10 μl of CCK-8 solution was added to each well and incubated for 2 h, and the absorbance was measured at 450 nm using a microplate reader (Thermo Fisher Scientific, U.S.A.).

### Quantitative reverse transcription-polymerase chain reaction analysis

Total RNA was extracted from NP cells using Trizol reagent (Invitrogen, U.S.A.) according to the manufacturer’s instructions, followed by RNA quality assessment using a NanoDrop 2000 spectrophotometer (Thermo Fisher Scientific, U.S.A.). Subsequently, the RNA was reverse-transcribed into cDNA using the PrimeScript RT reagent kit (Thermo Fisher Scientific, U.S.A.). Quantitative PCR (qPCR) analysis was performed using Power SYBR Green master mix (Life Technologies Inc., U.S.A.). The primer sequences used in the qPCR reactions were as follows: *ACSL1* (Forward: TGTTGACAAGCCAGAGAAG, Reverse: CTCGTTCCACCAGTTCAC); *GAPDH* (Forward: TCATTTCCTGGTATGACAACGA, Reverse: GTCTTACTCCTTGGAGGCC). The relative mRNA expression levels were calculated using the 2^−ΔΔCT^ method [21] with GAPDH as the internal reference. The qPCR thermal cycling conditions comprised an initial denaturation step at 95°C for 30 s, followed by 40 cycles of denaturation at 95°C for 10 s and annealing/extension at 60°C for 30 s.

### Western blotting

We extracted total proteins from NP cells and NP tissue from rats using 10% SDS-PAGE and transferred them onto a PVDF membrane. After washing with PBS, the membrane was blocked with 5% nonfat milk and then incubated with specific primary antibodies diluted as per the manufacturer’s recommendations overnight at 4°C. Subsequently, the membrane was incubated with secondary antibodies for 2 h at room temperature. Protein signals were visualized using the Pierce™ ECL Plus Western Blotting Substrate. β-actin was utilized as an internal control for normalization.

### Detection of intracellular ROS

The DCFH-DA probe (S0033s, Beyotime, China) was diluted 1:1000 in serum-free culture medium to achieve a final concentration of 10 µM. NP cells were then suspended in the diluted DCFH-DA solution at a concentration of 1 × 10^6^ cells/ml and incubated at 37°C for 20 min. Subsequently, cells were incubated with 10 μg/ml DAPI solution at 37°C for 15 min. Finally, cells were supplemented with serum-free culture medium. Fluorescence images were captured using a laser scanning confocal microscope (Zeiss LSM900).

### Mitochondrial membrane potential and apoptosis assays

The mitochondrial membrane potential was detected using the JC-1 fluorescence probe (C2006, Beyotime, China). According to the manufacturer’s instructions, JC-1 was diluted in serum-free medium at a ratio of 1:500 to a final concentration of 10 μg/ml. After incubation at 37°C in a cell culture incubator for 20 min, cells were inverted every 3–5 min to ensure complete probe–cell contact. Subsequently, cells were washed three times with serum-free medium. Following staining as directed by the manufacturer, analysis was conducted using flow cytometry with the FITC channel to measure the fluorescence intensity of JC-1 aggregates and monomers.

The dual-staining method with FITC and propidium iodide (PI) was employed to detect early cell apoptosis using flow cytometry. Briefly, NP cells were harvested, resuspended in binding buffer, and stained with FITC-conjugated annexin V and PI. After incubation, stained cells were analyzed using a flow cytometer, calibrated with control samples. Data analysis was performed to quantify apoptotic cells based on FITC and PI fluorescence profiles.

### Rat caudal intervertebral disc puncture model

Male Sprague-Dawley rats, approximately eight weeks of age, were anesthetized by intraperitoneal injection of 0.3% sodium pentobarbital solution at a dose of 1.0 ml/100 g body weight. The rats were placed in a supine position on a modeling platform, and their tails were disinfected three times with saline, iodine solution, and 75% ethanol. The caudal intervertebral discs were located, with Co5/6 designated as the experimental segment. A 20 G sterile solid needle was vertically inserted into the caudal intervertebral disc, followed by a single rotation to disrupt the NP and induce IVDD. The needle remained in place for 1 min. Four weeks after surgery, the rats were killed by cervical dislocation. Their tails were collected, stripped of adjacent soft tissues, and the vertebrae were fixed in 4% paraformaldehyde. All animal experiments were conducted at the Animal Center of Fujian Medical University. All experimental procedures involving animals complied with The Animal Research: Reporting of *in vivo* Experiments (ARRIVE) guidelines. The study was approved by the Ethics Committee for Laboratory Animals of Fujian Medical University (Approval No. IACUC FJMU 2024-0052).

### Tissue section staining

Paraffin-embedded tissue sections were stained using Safranin O-fast green and hematoxylin and eosin (H&E) methods. Immunofluorescence staining was performed on intervertebral disc sections with antibodies against ACSL1 (13989-1-AP, Proteintech), and Cy3-conjugated secondary antibodies were used for detection.

### Statistical analysis

The data were analyzed using R 4.1.0 and GraphPad Prism 10.0 software. Spearman’s correlation coefficient was employed to assess the correlation between continuous variables. Differences among three or more groups were evaluated using a one-way analysis of variance, while differences between two groups were assessed using a *t*-test followed by Tukey’s test. To address the issue of multiple testing, the FDR correction was applied to reduce the risk of false positives. Statistical significance was determined at a significance level of *P* value less than 0.05.

## Results

### The crucial modules associated with IVDD using WGCNA

**Figure 2:**
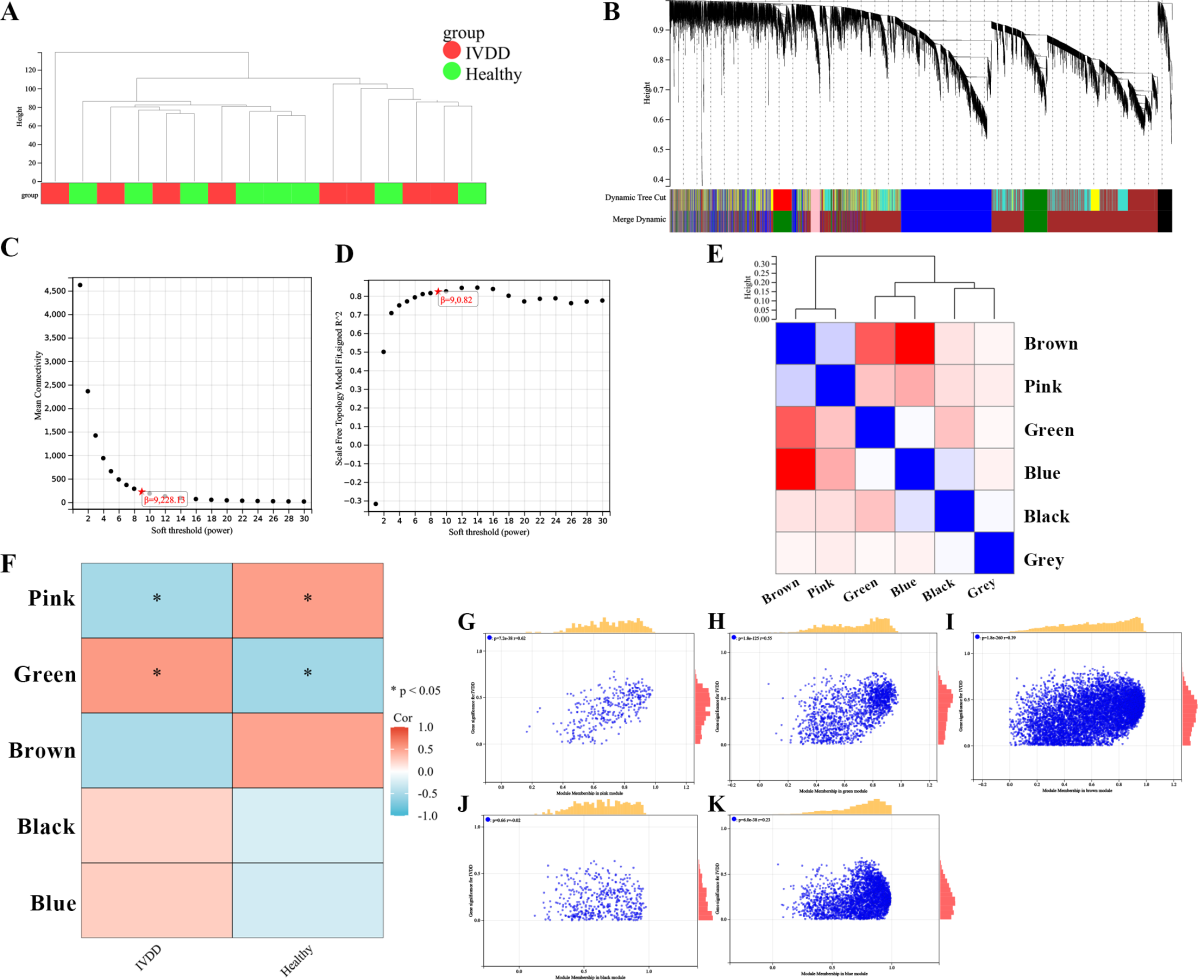
Identification of significant modules using WGCNA and construction of weighted co-expression network. (**A**) Genetic tree diagram. (**B**) Clustering dendrogram depicting clinical data from 16 samples. (**C,D**) Exploring scale-free fit index and mean connectivity across different soft-thresholding powers β. (**E**) Clustering heat map of module feature vectors. (**F**) Correlation heat map between MEs and clinical characteristics of IVDD patients, showing correlation coefficients and *P* values in each cell. (**G–K**) Scatter plots of the GS score and MM for genes in the four modules. *P < 0.05. GS, gene significance; IVDD, intervertebral disc degeneration; MEs, module eigengenes; MM, module membership; WGCNA, weighted gene co-expression network analysis.

We constructed a co-expression network using the R package WGCNA in combination with clinical data (Figure 2A). Cluster evaluation was performed on the 16 samples to assess data quality. We applied a height cutoff of 200, sensitivity of 3, and module merging threshold of 0.25 to identify and eliminate nonviable outliers (Figure 2B). Using the pick soft threshold function, we determined the optimal soft threshold for this model to be 9, resulting in an *R*^2^ value of 0.82 and a mean connectivity of 228.13 (Figure 2C and D). Genes with similar expression patterns were grouped into six co-expression modules: pink, green, brown, black, blue, and gray (Figure 2E). The gray module, consisting of 434 genes, was excluded because it could not be clearly associated with any specific functional module. Co-expression network analysis was conducted to identify gene sets highly correlated with IVDD patients (Figure 2F). For each module, the eigengene (first principal component) was calculated to represent the overall expression profile. The correlation between MEs and gene significance (GS) was then assessed using Pearson’s correlation coefficient, as shown in Figure 2G–K. The characteristic genes of the green module exhibited a significant positive correlation with IVDD (correlation coefficient = 0.55, *P* = 0.03), while the pink module showed a strong negative correlation (correlation coefficient = −0.53, *P* = 0.03). These results suggest that the green module may play a role in IVDD development, while the pink module may offer a protective effect. Further analysis of the MEs confirmed a significant correlation between gene module membership and gene significance (GS ratings) in both the pink and green modules (Figure 2G–K).

### Functional enrichment analysis identifies key pathways enriched in the MEs

**Figure 3:**
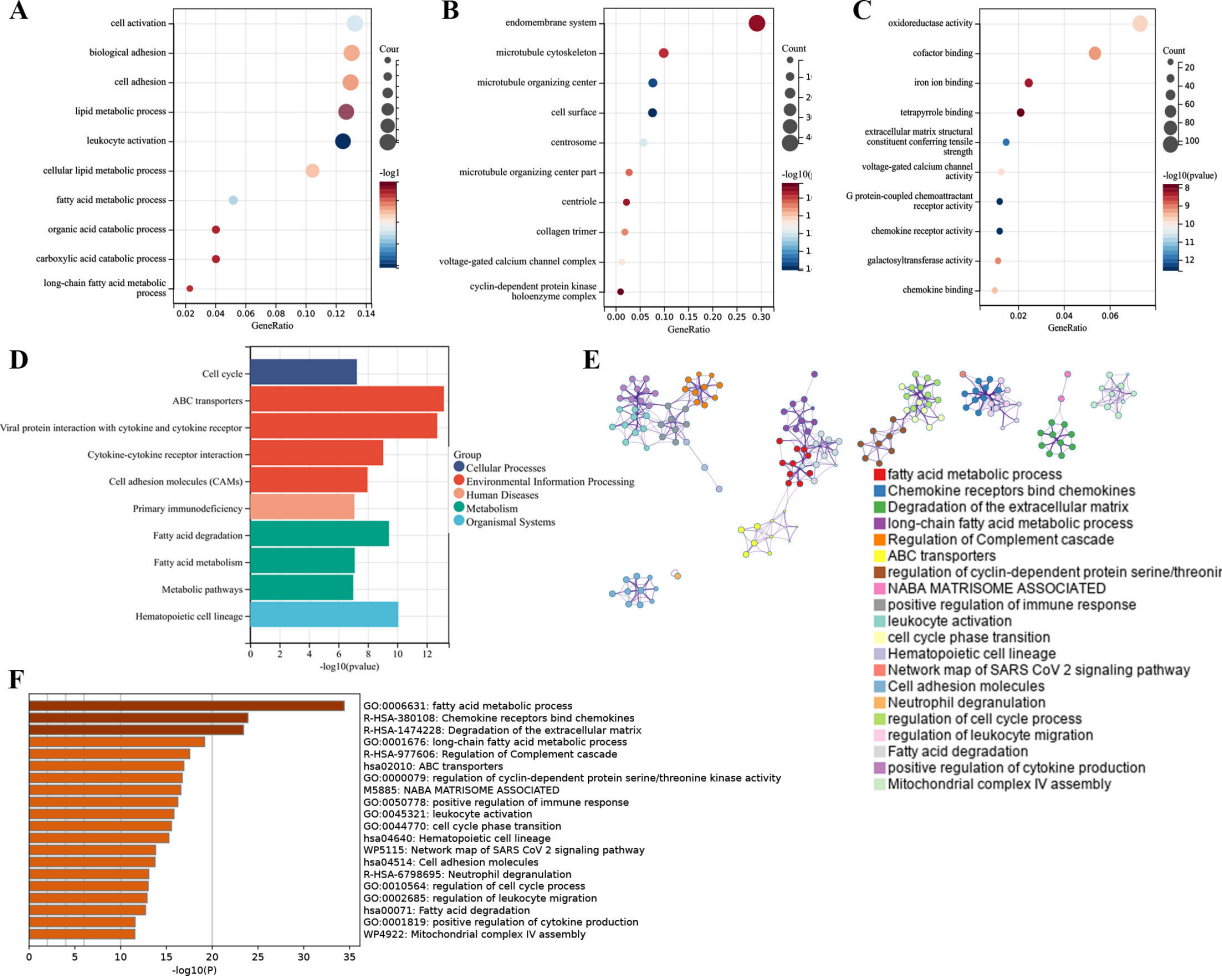
Functional enrichment analysis of MEs. (**A–C**) GO analysis of biological processes (**A**), cellular components (**B**), and molecular functions (**C**). (**D**) KEGG enrichment analysis of the MEs. (**E,F**) Metascape analysis. GO, Gene Ontology; KEGG, Kyoto Encyclopedia of Genes and Genomes; MEs, module eigengenes.

To gain insights into the pathways enriched by MEs, we performed functional enrichment analysis (including GO, KEGG, and Metascape analyses) specifically on the pink and green modules, which were selected based on their biological relevance and significance in the context of our study. GO analysis revealed significant enrichment of MEs in several biological processes, including cell activation, biological adhesion, lipid metabolic processes, and leukocyte activation (Figure 3A). Additionally, MEs were predominantly enriched in the endomembrane system in terms of cellular components (Figure 3B), and in oxidoreductase activity, cofactor binding, and iron ion binding in terms of molecular functions (Figure 3C). These results suggest potential functional roles of MEs in lipid metabolism, iron ion binding, and immune activation. Furthermore, KEGG enrichment analysis confirmed the association of MEs with lipid metabolism and immune-related pathways (Figure 3D). Additionally, utilizing the online tool Metascape, we further analyzed and visualized the interactions within the MEs, revealing enrichment in fatty acid metabolism, chemokine receptors binding chemokines, and degradation of the extracellular matrix (Figure 3E and F). These findings provide important insights into the roles of these genes in lipid metabolism and immune function.

### Association of differentially expressed genes with immune response in IVDD patients

**Figure 4:**
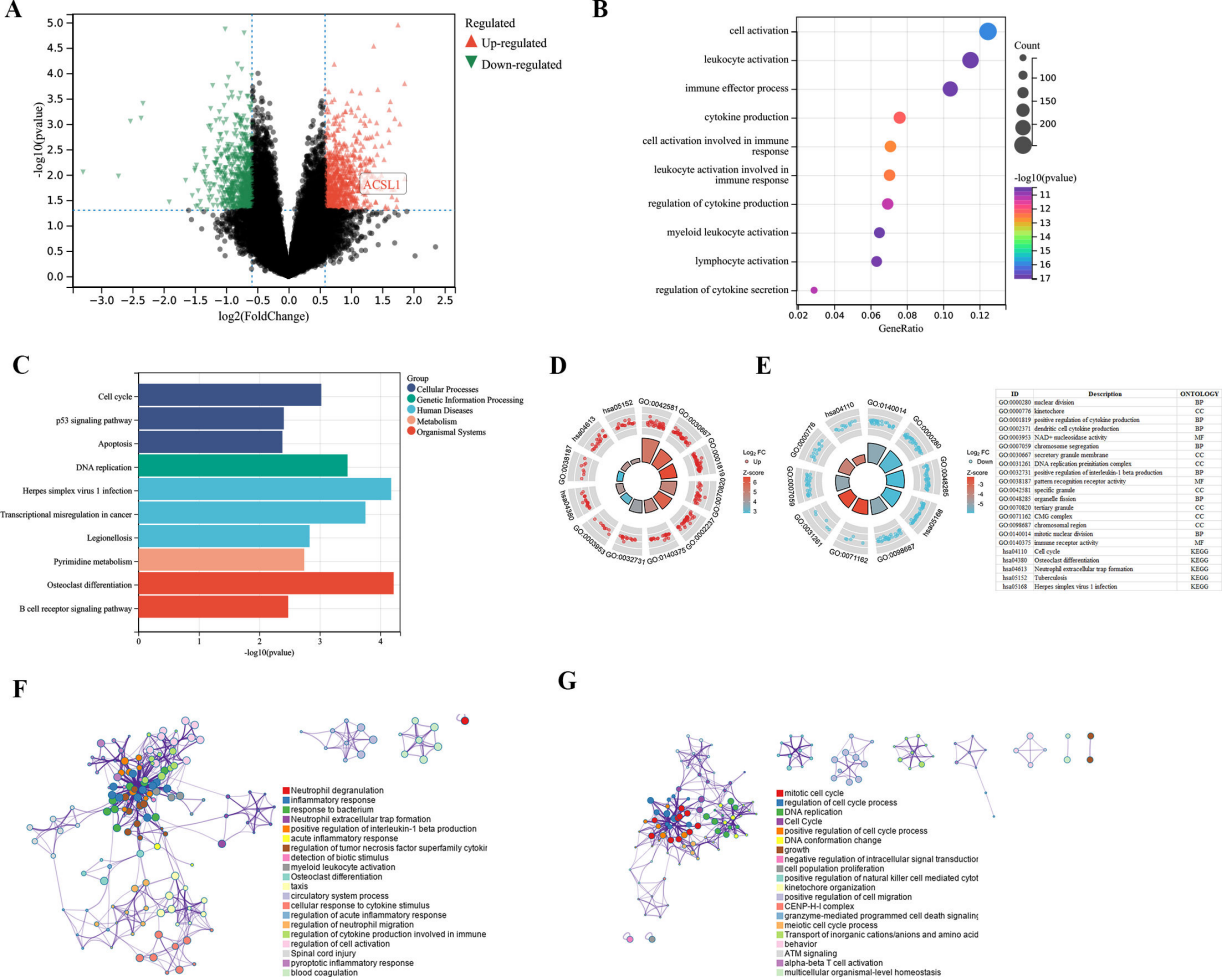
DEGs were associated with immune response. (**A**) Volcano plot depicting DEGs, among which *ACSL1* is up-regulated in IVDD samples. (**B**) GO enrichment analysis of DEGs. (**C**) KEGG enrichment analysis of DEGs. (**D,E**) GO and KEGG combined functional enrichment analysis of up-regulated (**D**) and down-regulated (**E**) DEGs. (**F,G**) Metascape analysis of up-regulated DEGs (**F**) and down-regulated DEGs (**G**). DEGs, differentially expressed genes; GO, Gene Ontology; KEGG, Kyoto Encyclopedia of Genes and Genomes.

Gene differential expression analysis revealed 725 up-regulated and 686 down-regulated genes in IVDD patients (Figure 4A, Table S6). To explore the potential functions of these DEGs, we conducted GO and KEGG enrichment analyses. GO analysis showed that DEGs were primarily associated with cell activation, leukocyte activation, and immune effector processes (Figure 4B). KEGG analysis highlighted the enrichment of DEGs in pathways related to cell cycle, p53 signaling, and apoptosis (Figure 4C). Further analysis involved GO and KEGG logFC combined analysis using the R package clusterProfiler on up-regulated and down-regulated DEGs. Up-regulated DEGs were notably enriched in specific granule, secretory granule membrane, and positive regulation of cytokine production (Figure 4D), suggesting a link to immune cell degranulation. Conversely, down-regulated DEGs were mainly enriched in processes such as mitotic nuclear division, nuclear division, and organelle fission (Figure 4E), indicating a potential role in promoting cell proliferation to delay IVDD progression. The above findings are consistent with the perspectives of Silwal P et al. [22]. Notably, metascape analysis of DEGs revealed enrichment in processes related to positive regulation of cytokine production, neutrophil functions, and regulation of inflammatory response (Figure 4F and G). These findings suggest that gene alterations may contribute to IVDD development by influencing immune responses and cell proliferation regulation.

### Immune analysis reveals the immunological landscape of intervertebral degenerative disc microenvironment

**Figure 5:**
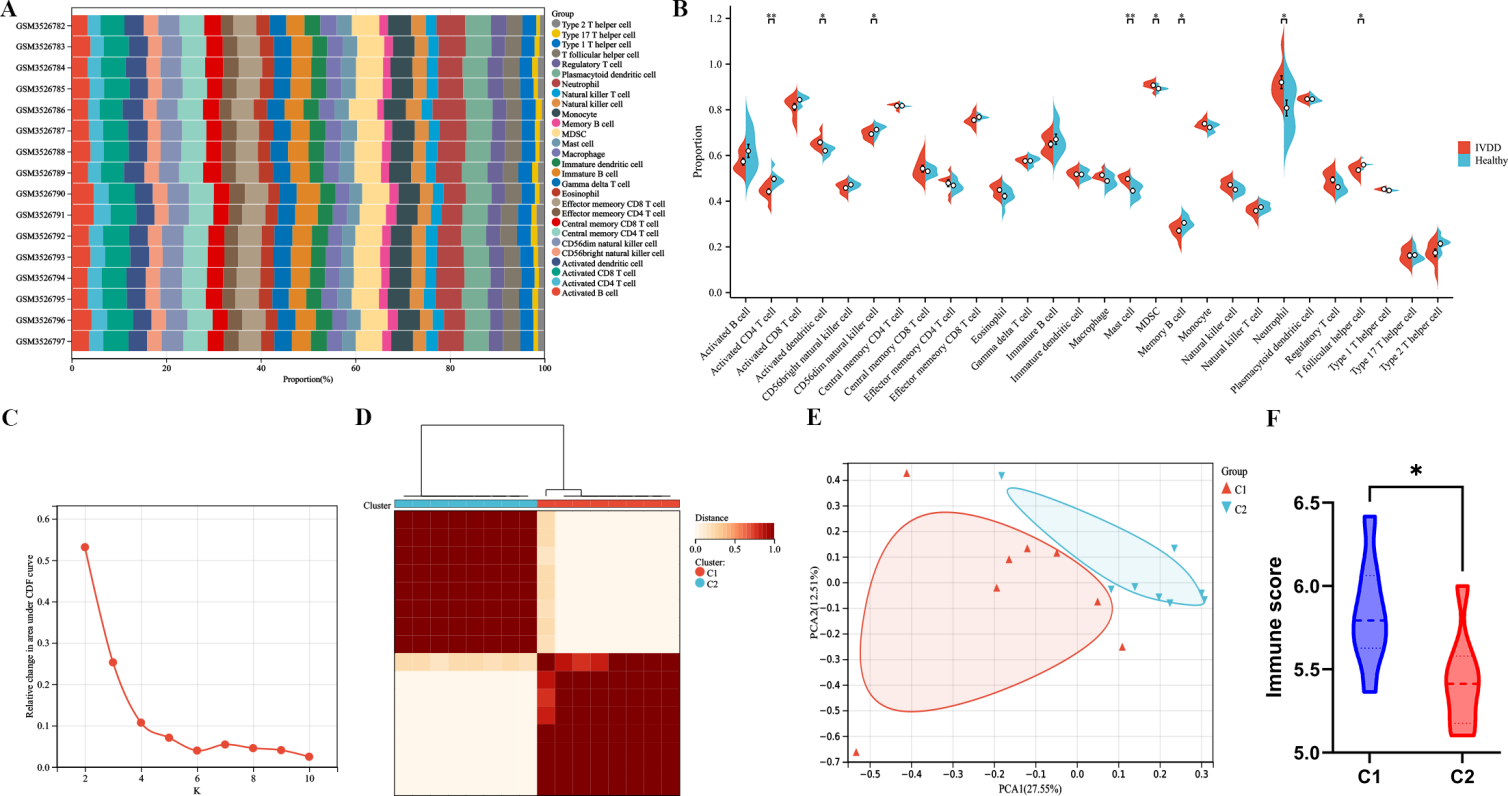
Immunological landscape of degenerative intervertebral disc. (**A**) The stacked bar chart illustrates the immune cell infiltration across different samples. (**B**) Comparison of immune cell infiltration profiles between intervertebral disc degeneration and healthy samples across 28 different types of immune cells. (**C**) Relative change in area under CDF curve. (**D**) Consensus clustering in the ‘ConsensusClusterPlus’ package was used to identify different immune subtypes. (**E**) Principal component analysis of the different immune subtypes. (**F**) Comparison of the immune score C1 and C2 subtypes. *P < 0.05, **P < 0.01.

Extensive studies have demonstrated an association between immune cell infiltration in the intervertebral disc microenvironment and the progression of disc degeneration [23,24]. To explore the immunological landscape of degenerated intervertebral discs, we first employed ssGSEA to assess the levels of 28 different immune cell infiltrates in various samples (Table S2). As depicted in Figure 5A and B, activated dendritic cells, mast cells, myeloid-derived suppressor cells, and neutrophils exhibited significant infiltration in IVDD samples, particularly highlighting the presence of neutrophils. Subsequently, to distinguish immune subtypes of IVDD, we performed consensus clustering analysis on 16 samples using an immunological gene dataset sourced from ImmPort (Table S3). Figure 5C and D illustrates the samples classified into two clusters: C1 and C2. Additionally, principal component analysis demonstrated effective sample differentiation between the two clusters (Figure 5E). To further quantify the strength of immune responses between different clusters, we applied ssGSEA to calculate immune scores for different clusters (Table S4). The results revealed that C1 exhibited higher immune scores compared with C2 (Figure 5F), indicating a stronger immune infiltration level and immune response in C1.

### Identification and diagnostic significance of ferroptosis-related hub genes in IVDD

**Figure 6:**
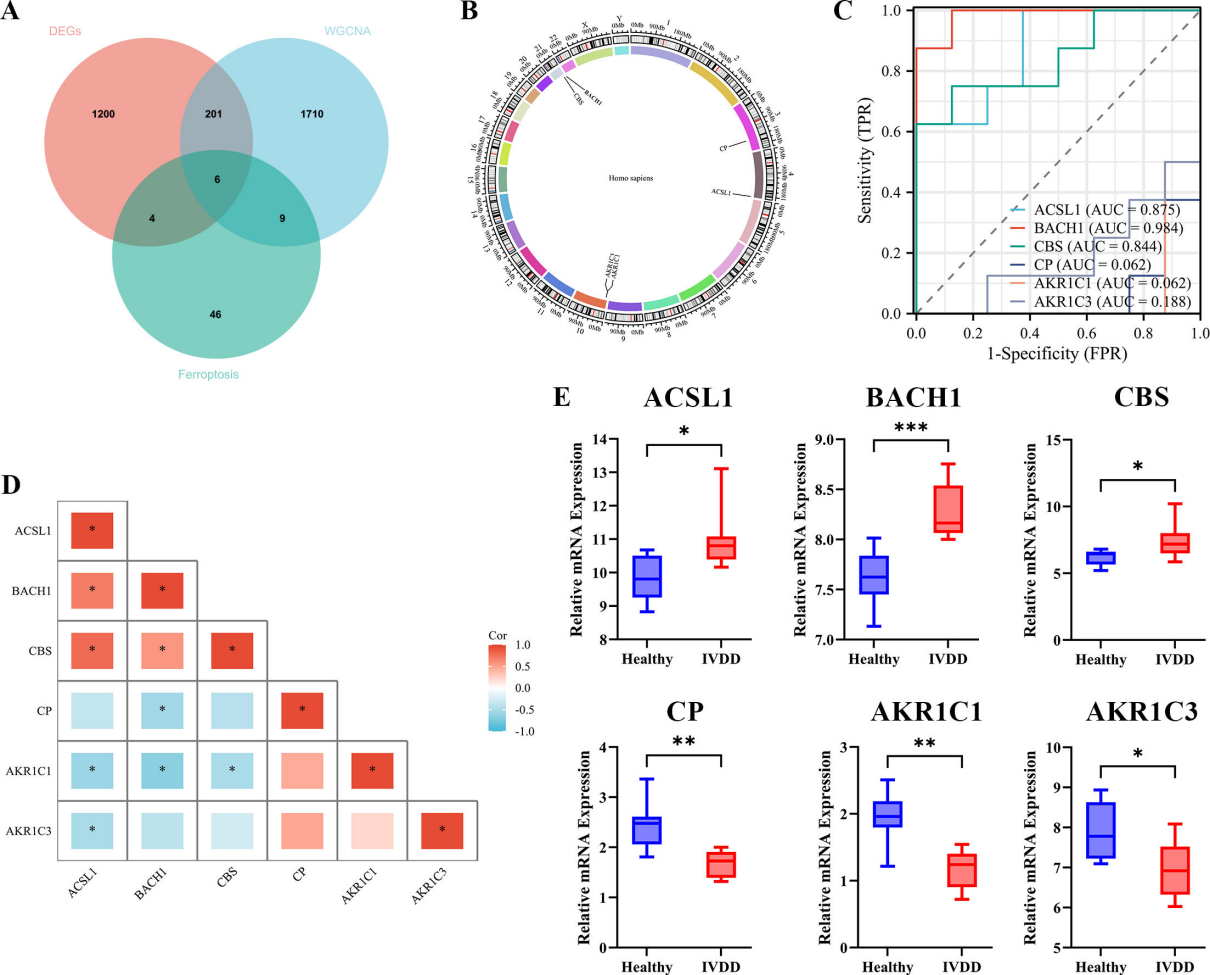
Identification of six hub genes in IVDD samples. (**A**) Venn diagram shows the intersection between DEGs, MEs from WGCNA, and ferroptosis-related genes. (**B**) Chromosomal co-ordinates of the six hub genes. (**C**) Diagnostic receiver operating characteristic analysis of six hub genes. (**D**) Heat map reveals the relationships among the six hub genes. (**E**) Relative expression level of six hub genes in healthy and IVDD samples. *P < 0.05, **P < 0.01, ***P < 0.001. DEGs, differentially expressed genes; IVDD, intervertebral disc degeneration; MEs, module eigengenes; WGCNA, weighted gene co-expression network analysis.

The ferroptosis of NP cells is closely associated with the occurrence and progression of IVDD [25]. To identify the key genes involved, we determined the hub genes by intersecting DEGs, MEs (pink and green modules), and ferroptosis-related genes. This analysis yielded six hub genes: *ACSL1*, *BACH1*, *CBS*, *CP*, *AKR1C1*, and *AKR1C3* (Figure 6A). The chromosome ideogram depicts the chromosomal locations of six hub genes (Figure 6B). Chromosomal co-ordinates of these hub genes are presented in Figure 5B. Diagnostic receiver operating characteristic analysis demonstrated that *ACSL1* (AUC: 0.875), *BACH1* (AUC: 0.984), and *CBS* (AUC: 0.844) exhibited high diagnostic value (Figure 6C). Correlation analysis of these hub genes was conducted and visualized using the ggplot2 package (Figure 6D). Additionally, in IVDD patients, the expression of *ACSL1*, *BACH1*, and *CBS* increased, while the expression of *CP*, *AKR1C1*, and *AKR1C3* decreased (Figure 6E, Figure S2).

### Identification of *ACSL1* as a biomarker of IVDD

**Figure 7:**
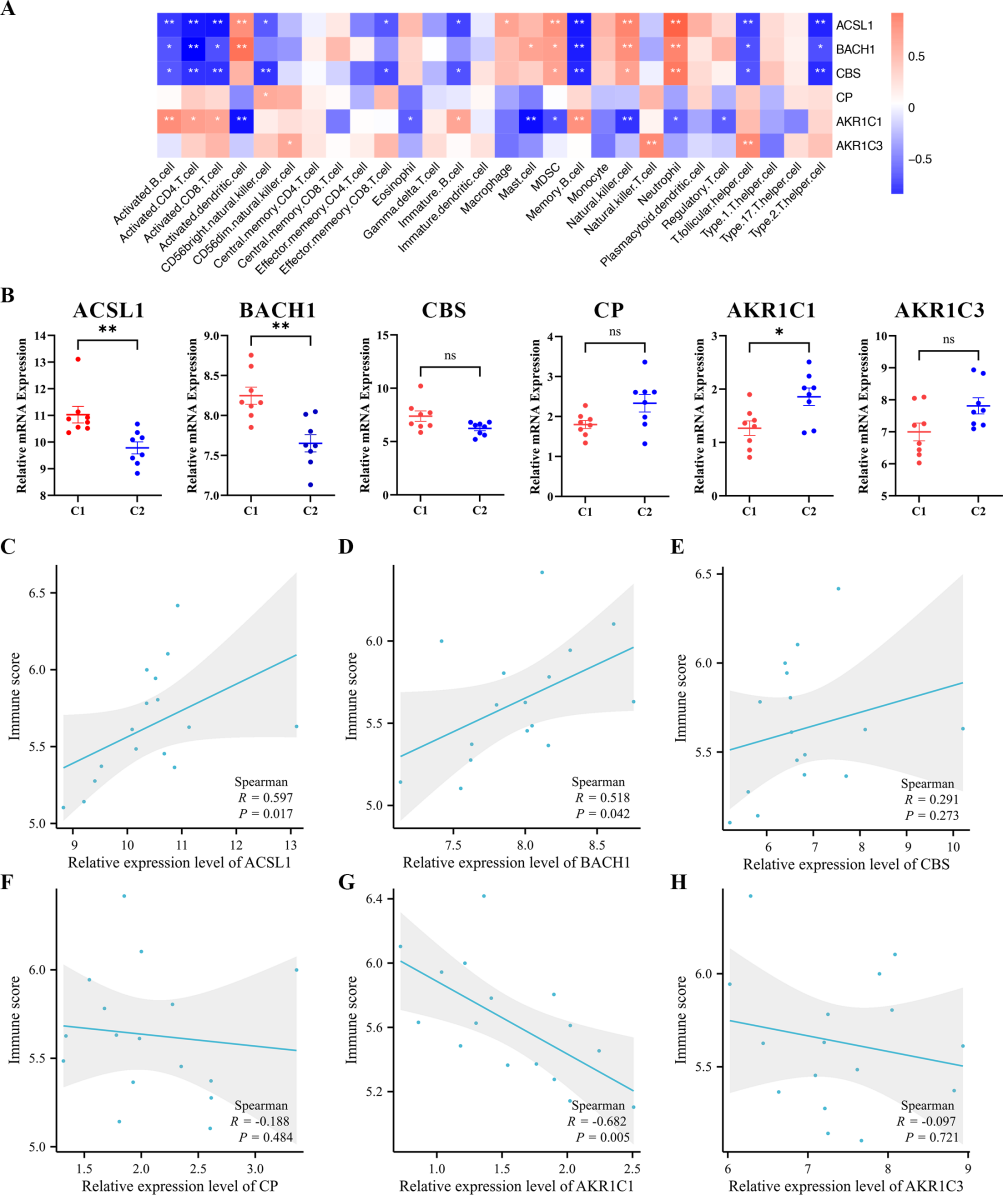
Identification of *ACSL1* as a biomarker of intervertebral disc degeneration. (**A**) Heat map shows the relationship between the expression level of hub genes and infiltration level of different immune cells. (**B**) Relative expression level of the hub genes between different immune subtypes. (**C–H**) Scatter plot showing the correlation between expression of hub genes and immune scores. *P < 0.05, **P < 0.01.

Given the known association between immune cell infiltration and IVDD progression [26,27], we conducted a co-expression analysis of hub genes with 28 different immune cell infiltrations. The results, visualized using the ggplot2 package (Figure 7A, Table S5), showed that *ACSL1* expression correlates with 14 types of immune cell infiltrations. Moreover, *ACSL1* and *BACH1* were found to be up-regulated in the C1 subtype, while *AKR1C1* was elevated in the C2 subtype (Figure 7B). This suggests that *ACSL1* and *BACH1* may promote immune cell infiltration, while *AKR1C1* may inhibit it. To validate these findings, we analyzed the correlation between hub gene expression levels and immune scores (Figure 7C–H). The strongest correlation was observed between *ACSL1* and immune scores (*R* = 0.597, *P* = 0.017). Therefore, *ACSL1* emerges as a potential biomarker for IVDD, warranting further investigation into its underlying mechanisms.

### *ACSL1* was up-regulated in IL-1β-induced degenerative NP cell

**Figure 8:**
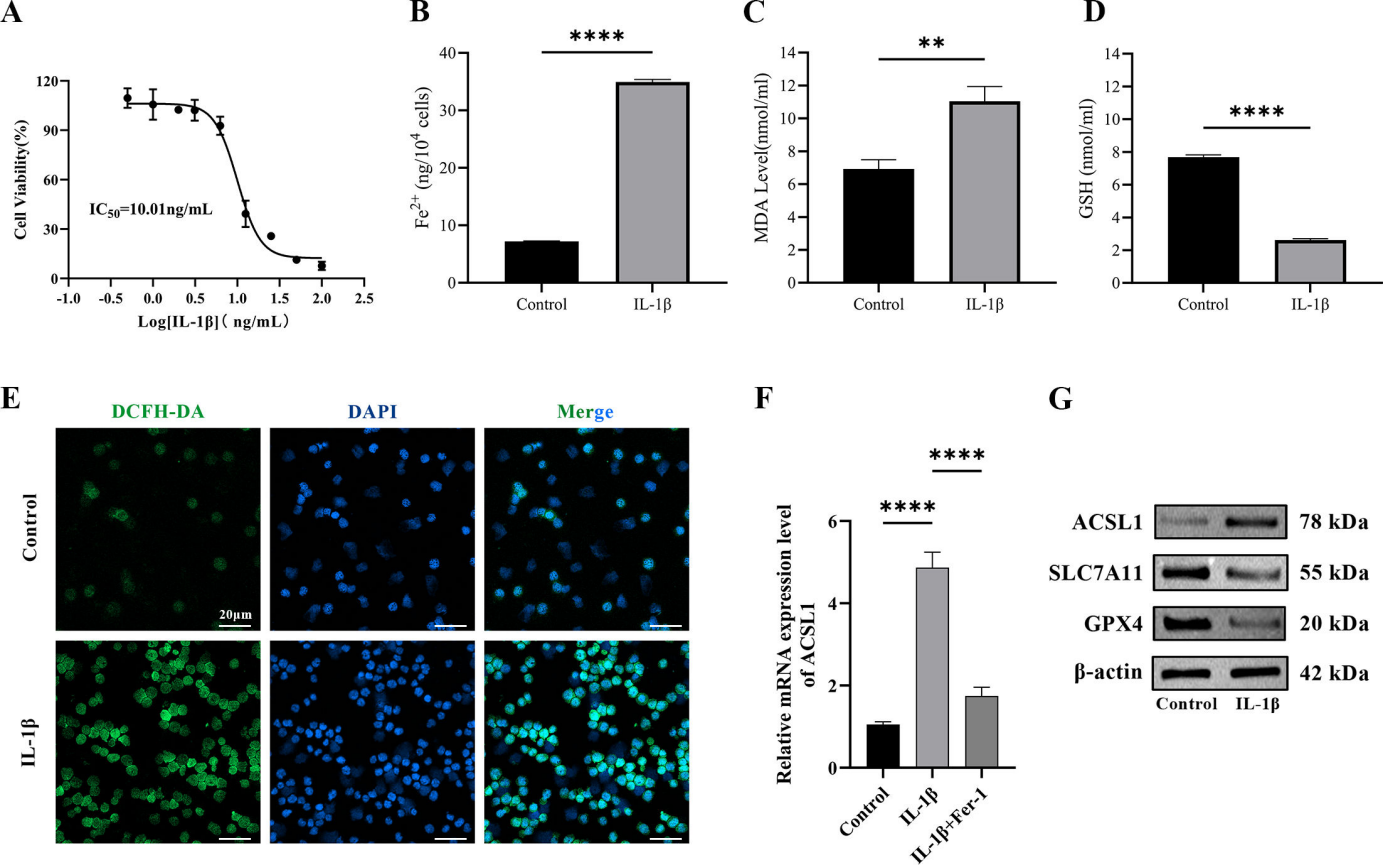
Validation of *ACSL1* changes in IL-1β-induced degenerative NP cell. (**A**) IC_50_ of IL-1β on NP cells. (**B–D**) Fe^2+^ concentration, MDA level and GSH level in different groups. (**E**) Immunofluorescent staining showed ROS level in IL-1β-treated group and control group (scale bar: 20 μm). (**F**) Relative mRNA expression of *ACSL1* in different groups. (**G**) ACSL1, GPX4, and SLC7A11 protein expression of NP cells in different groups. *P < 0.05, **P < 0.01, ***P < 0.001, ****P < 0.0001. GSH, glutathione; MDA, malondialdehyde; NP, nucleus pulposus; ROS, reactive oxygen species.

In order to validate the expression level of *ACSL1 in vitro*, we established an IL-1β-induced degenerative NP cell model. The IC_50_ of IL-1β on NP cells was determined to be 10.01 ng/ml (Figure 8A). In this model, compared with the control group, the IL-1β-treated group exhibited higher concentrations of Fe^2+^ and MDA, as well as decreased levels of GSH content (Figure 8B–D). Additionally, IL-1β treatment led to elevated protein expression of ACSL1, accompanied by reduced expression of GPX4 and SLC7A11 proteins in NP cells (Figure 8G). Notably, the measurement of ROS levels using the DCFH-DA probe revealed significantly higher fluorescence intensity in the IL-1β-treated group compared with the control (Figure 8E), suggesting the occurrence of ferroptosis in NP cells stimulated by IL-1β. Furthermore, the relative mRNA expression level of *ACSL1* was up-regulated in the IL-1β-treated group compared with the control (Figure 8F). Interestingly, concurrent treatment of NP cells with IL-1β and Fer-1 resulted in reduced expression of *ACSL1* compared with the IL-1β-treated group alone (Figure 8F), indicating that ferroptosis inhibition could regulate *ACSL1* expression. Similarly, we observed increased *ACSL1* expression in the erastin-treated group compared with the control ( Figure S1), further supporting a close association between *ACSL1* expression in NP cells and the occurrence of ferroptosis. These findings collectively affirm the up-regulation of *ACSL1* expression in the IL-1β-induced degenerative NP cell model.

### Silencing of *ACSL1* attenuates ferroptosis in degenerative NP cell

**Figure 9:**
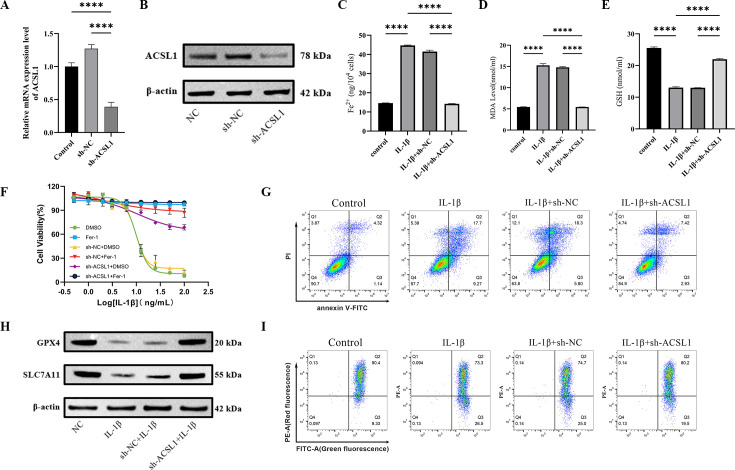
Knockdown of *ACSL1* ameliorates ferroptosis in degenerative NP cells. (**A,B**) The interfering efficiency of *ACSL1* in NP cells was evaluated by RT-qPCR and western blot. (**C–E**) The intracellular Fe^2+^ concentration, MDA, and GSH levels of NP cells in different groups. (**F**) Cell viability in different groups detected by CCK-8 assay. (**G**) Apoptosis levels of NP cells in different groups using flow cytometry. (**H**) GPX4 and SLC7A11 protein expression of NP cells in different groups. (**I**) Mitochondrial membrane potential of NP cells in different groups detected by JC-1 probe using flow cytometry. *P < 0.05, **P < 0.01, ***P < 0.001, ****P < 0.0001. GSH, glutathione; MDA, malondialdehyde; NP, nucleus pulposus; RT-qPCR, quantitative reverse transcription polymerase chain reaction.

To investigate the impact of *ACSL1* reduction in ferroptosis in NP cells, we utilized sh-RNA transfection to down-regulate *ACSL1* expression. The efficacy of *ACSL1* transfection interference was confirmed through RT-qPCR and western blot analysis (Figure 9A and B). Subsequently, we evaluated the Fe^2+^ concentration, as well as the levels of MDA and GSH in NP cells subjected to different treatments. Our findings demonstrated that *ACSL1* knockdown resulted in decreased Fe^2+^ concentration and MDA levels, while concurrently increasing the GSH level in IL-1β-treated NP cells (Figure 9C–E). Moreover, we assessed cell viability using a CCK-8 assay. The results indicate that the use of the ferroptosis inhibitor Fer-1 can alleviate the reduction in cell viability induced by IL-1β. Similarly, knockdown of *ACSL1* also partially mitigates the decrease in NP cell viability caused by IL-1β (Figure 9F). Additionally, IL-1β treatment induced elevated NP cell apoptosis, a response that was reversed by *ACSL1* knockdown (Figure 9G). Furthermore, Figure 9I illustrates that IL-1β treatment reduced the mitochondrial membrane potential in NP cells, whereas knockdown of *ACSL1* rescued this adverse effect induced by IL-1β. Collectively, these findings highlight the efficacy of *ACSL1* knockdown in mitigating ferroptosis in degenerative NP cells.

### *ACSL1* elevation in IVDD rat model

**Figure 10:**
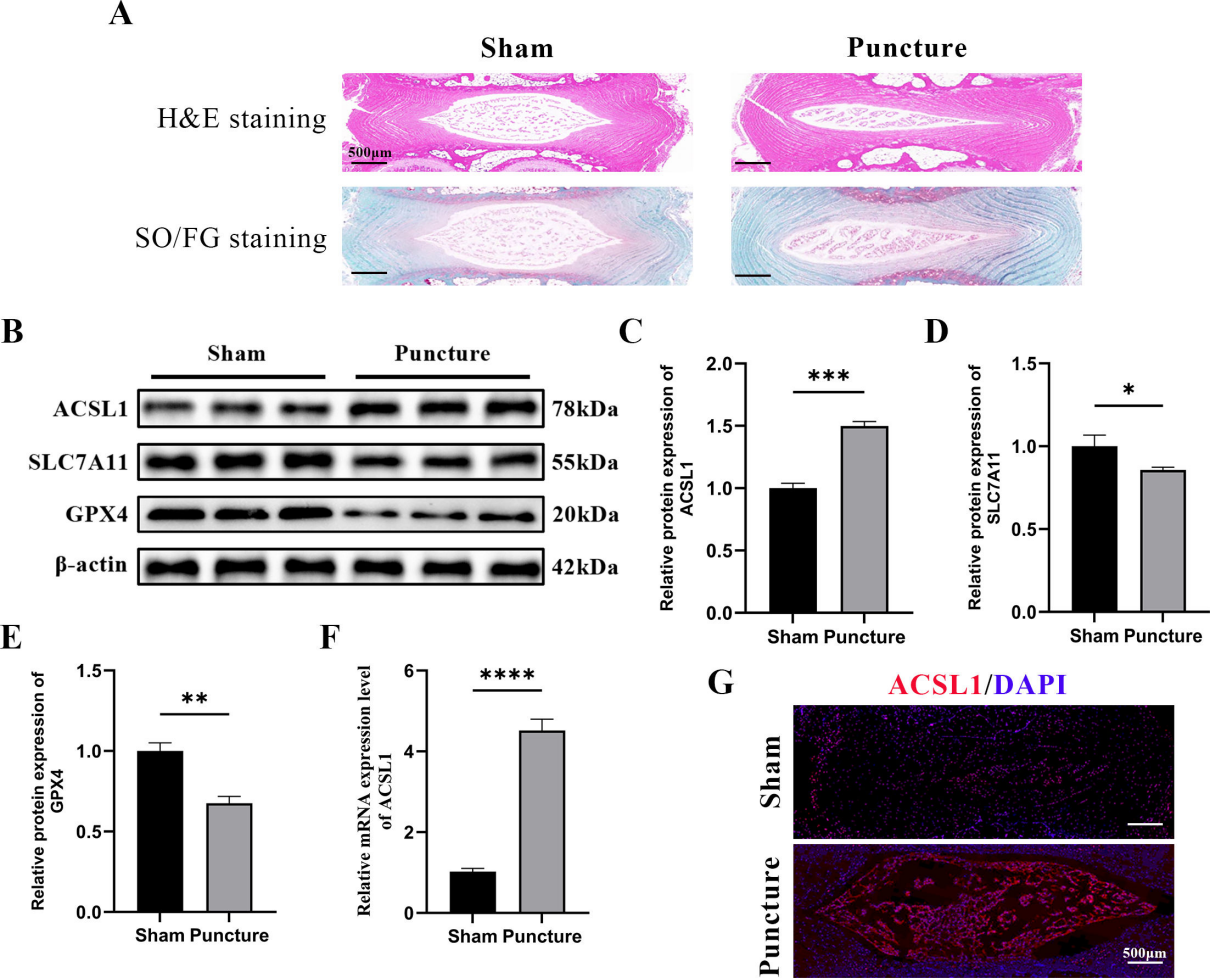
*ACSL1* elevation in IVDD rat model. (**A**) H&E staining and SO/FG staining of rat disc tissue. (**B–E**) The western blot result revealed that the expression of *ACSL1* was increased in IVDD rats, while SLC7A11 and GPX4 were decreased. (**F**) RT-qPCR revealed that *ACSL1* mRNA elevated in IVDD rats. (**G**) Immunofluorescence staining of *ACSL1* in rat disc tissue. H&E, hematoxylin and eosin; IVDD, intervertebral disc degeneration; RT-qPCR, quantitative reverse transcription polymerase chain reaction.

To validate the *in vivo* changes in *ACSL1* expression in the NP of IVDD rats, we established a puncture-induced IVDD model at the Co5/6 level. As shown in Figure 10A, disc height significantly decreased in the puncture group, consistent with the characteristics of IVDD. Western blot analysis revealed elevated *ACSL1* expression in the NP tissue of the IVDD group, while SLC7A11 and GPX4 expression levels were reduced (Figure 10B–E). qPCR analysis further confirmed increased *ACSL1* mRNA expression in the NP of the IVDD group (Figure 10F). Additionally, immunofluorescence staining of disc tissue showed a higher *ACSL1* fluorescence intensity in the IVDD group (Figure 10G). These findings align with our *in vitro* results, further supporting *ACSL1* as a potential biomarker for IVDD.

## Discussion

The pivotal role of ferroptosis in IVDD has recently garnered widespread attention [28,29]. Ferroptosis represents an iron-dependent form of cell death primarily driven by lipid peroxidation [30]. The present study utilized WGCNA to identify MEs most relevant to IVDD. Functional enrichment analysis revealed that these MEs were primarily enriched in pathways associated with lipid metabolism and immune responses (Figure 3). Subsequent differential expression analysis of transcriptome data identified up-regulation of *ACSL1* in IVDD samples, with increased expression levels (Figure 4). Additionally, we combined data from three GEO datasets: GSE23130, GSE153761, and GSE167199, all of which contain intervertebral disc tissue samples. Our analysis of *ACSL1* expression revealed that it remains significantly elevated in the IVDD group compared with the control group, aligning with our previous findings (Figure S2). Furthermore, functional enrichment analysis of DEGs also indicated that up-regulated DEGs were associated with immune responses in IVDD (Figure 4). The microenvironment of a healthy intervertebral disc is characterized by the presence of a blood–NP barrier [31], which restricts immune cell infiltration, maintaining minimal levels within the disc. However, as disc degeneration advances, microvessels penetrate the disc through ruptured cartilage endplates, facilitating the ingress of immune cells, notably macrophages and neutrophils [8]. These infiltrating immune cells not only recruit additional immune cells by secreting chemokines [23] but also contribute to extracellular matrix degradation and NP cell death by releasing inflammatory cytokines such as IL-1β, IL-17, and TNF [32,33], as well as through mechanisms such as M1 macrophage polarization [13]. It has been reported that ferroptosis is closely related to immune cell infiltration in the tumor microenvironment [34]. To explore the immune profile in degenerative intervertebral disc, we conducted immune scoring and consensus clustering analysis on all samples. The results revealed that subtype C1 exhibited higher immune scores compared with C2 (Figure 5). Additionally, *ACSL1* expression was found to be elevated in the C1 subtype (Figure 7). Therefore, it is hypothesized that *ACSL1* is highly correlated with the immune landscape of IVDD. As expected, on one hand, *ACSL1* expression correlated with the degree of infiltration of 14 immune cell types; on the other hand, *ACSL1* expression levels were highly correlated with immune scores of the samples (Figure 7). However, our study used peripheral blood samples rather than disc tissue samples, meaning that the immune cell infiltration data reflect systemic immune profiles and not actual infiltration into the intervertebral disc. This approach may not fully capture the immune dynamics within the disc itself. Future research should use direct disc tissue samples to better understand IVDD mechanisms. Despite these limitations, we believe *ACSL1* has potential as a biomarker for IVDD. To address the small sample size and the use of peripheral blood samples, we further validated *ACSL1*’s significance with intervertebral disc tissue datasets (Figure S2) and through additional *in vitro* and *in vivo* experiments.

*ACSL1*, a member of the acyl-CoA synthetase long-chain family, has been demonstrated to participate in the activation and downstream metabolism or synthesis processes of fatty acids [35]. The ACSL enzyme family is capable of activating polyunsaturated fatty acids (PUFA) into PUFA-PLs, thereby inducing ferroptosis in cells11. However, the effects mediated by *ACSL1* in cellular ferroptosis are multifaceted. On one hand, *ACSL1* can confer resistance to ferroptosis in ovarian cancer by increasing the N-myristoylation and stability of FSP110. Conversely, up-regulation of *ACSL1* expression can promote sensitivity to ferroptosis in acute myeloid leukemia [12]. Nevertheless, the mechanistic role of *ACSL1* in the progression of IVDD remains unclear.

Iron overload, lipid peroxidation, and mitochondrial dysfunction are typical features distinguishing ferroptosis from other forms of cell death. Assessment of IL-1β effects on NP cells revealed a significant increase in iron overload and lipid peroxidation. Elevated Fe^2+^ and MDA levels and decreased GSH levels after IL-1β treatment further confirmed its ability to induce ferroptosis in NP cells (Figure 8). Additionally, we observed a decrease in the expression of ferroptosis-related proteins GPX4 and SLC7A11 (Figure 8), further supporting the occurrence of ferroptosis [36]. Moreover, intracellular ROS levels are an important indicator of ferroptosis [37]. In this study, we found a significant increase in ROS levels in NP cells after IL-1β treatment (Figure 8). Following IL-1β stimulation, the expression of *ACSL1* in NP cells increased (Figure 8). Similarly, after treatment with erastin, *ACSL1* expression also increased (Figure S1). Furthermore, this increase in *ACSL1* expression could be inhibited by the ferroptosis inhibitor Fer-1 (Figure 8), indicating that the up-regulation of *ACSL1* is induced by cellular ferroptosis rather than directly by IL-1β.

In our study, the observed changes in MDA, GSH, GPX4, and SLC7A11 levels provide compelling evidence for the involvement of ferroptosis in IVDD. The significant increase in MDA, a byproduct of lipid peroxidation, aligns with the hallmark of ferroptosis, indicating oxidative damage to cellular membranes. Concurrently, the depletion of GSH and down-regulation of GPX4 suggest a compromised antioxidant defense system, which is critical for preventing lipid peroxidation and subsequent ferroptosis. Furthermore, the reduced expression of SLC7A11, a key component of the cystine/glutamate antiporter system Xc−, underscores the disruption of cystine uptake and GSH synthesis, further exacerbating oxidative stress and ferroptosis. These findings collectively highlight the functional relevance of these molecules in ferroptosis and their potential roles in IVDD pathogenesis. Targeting these pathways, such as enhancing GPX4 activity or restoring SLC7A11 function, may offer novel therapeutic strategies for mitigating IVDD progression. Importantly, our experimental results further support the central role of *ACSL1* in regulating ferroptosis. In IL-1β-treated NP cells, *ACSL1* knockdown led to a significant reduction in Fe2+ and MDA levels, accompanied by a marked increase in GSH levels (Figure 9), suggesting a reversal of IL-1β-induced ferroptotic effects. Moreover, ACSL1 down-regulation improved cell viability and reduced apoptosis in IL-1β-treated NP cells (Figure 9). Notably, the protein levels of GPX4 and SLC7A11 were also up-regulated following *ACSL1* knockdown, further reinforcing the protective role of *ACSL1* inhibition against ferroptosis. These findings not only validate the functional relevance of *ACSL1* in ferroptosis regulation but also highlight its potential as a therapeutic target for alleviating IVDD progression.

Mitochondrial membrane potential serves as a crucial indicator of mitochondrial function, and during ferroptosis, mitochondrial damage is often observed [38]. Following IL-1β stimulation, a significant reduction in mitochondrial membrane potential was observed in NP cells. However, knocking down *ACSL1* reversed the decrease in mitochondrial membrane potential induced by IL-1β in NP cells (Figure 9). Thus, we speculate a close association between *ACSL1* expression levels and mitochondrial dysfunction during ferroptosis. In addition, *in vivo* experiments also revealed increased *ACSL1* expression in the intervertebral disc tissue of IVDD rats (Figure 10). In summary, knocking down *ACSL1* effectively rescues IL-1β-induced iron overload, lipid peroxidation, cell apoptosis, and mitochondrial dysfunction, underscoring the pivotal role of *ACSL1* in ferroptosis of degenerative NP cell.

Based on our findings, *ACSL1* shows potential as a biomarker for diagnosing IVDD. However, given the complexity of IVDD and the relatively small sample size used in this study, our conclusions should be interpreted with caution. Larger cohorts and additional studies are necessary to validate the role of *ACSL1* in IVDD. Moreover, while this study suggests a connection between *ACSL1* and ferroptosis in NP cells, the exact biological mechanisms through which *ACSL1* mediates ferroptosis remain unclear and require further exploration.

## Conclusions

*ACSL1* may serve as a biomarker for IVDD due to its association with ferroptosis in NP cells. However, the small sample size in this study highlights the need for further validation in larger cohorts to confirm these findings and understand the underlying mechanisms.

## Supplementary material

Supplementary Table 1

Supplementary Table 2

Supplementary Table 3

Supplementary Table 4

Supplementary Table 5

Supplementary Table 6

Supplementary Figure 1

Supplementary Figure 2

## Data Availability

Data are provided within the manuscript or supplementary information files (https://doi.org/10.5281/zenodo.13767458).

## Abbreviations

ACSL1: acyl-CoA synthetase long-chain family member 1
DEGs: differentially expressed genes
GSH: glutathione
IVDD: intervertebral disc degeneration
MDA: malondialdehyde
MEs: module eigengenes
NP: nucleus pulposus
ROS: reactive oxygen species
WGCNA: weighted gene co-expression network analysis
ssGSEA: single-sample gene set enrichment analysis
