# Supplementary figures and images for: Identification and functional validation of ACSL1 as a biomarker regulating ferroptosis in nucleus pulposus cell
Source: Biosci Rep. 2025 Apr 2;45(4):215–31. doi: 10.1042/BSR20241414 (PMC12203930; doi:10.1042/BSR20241414)

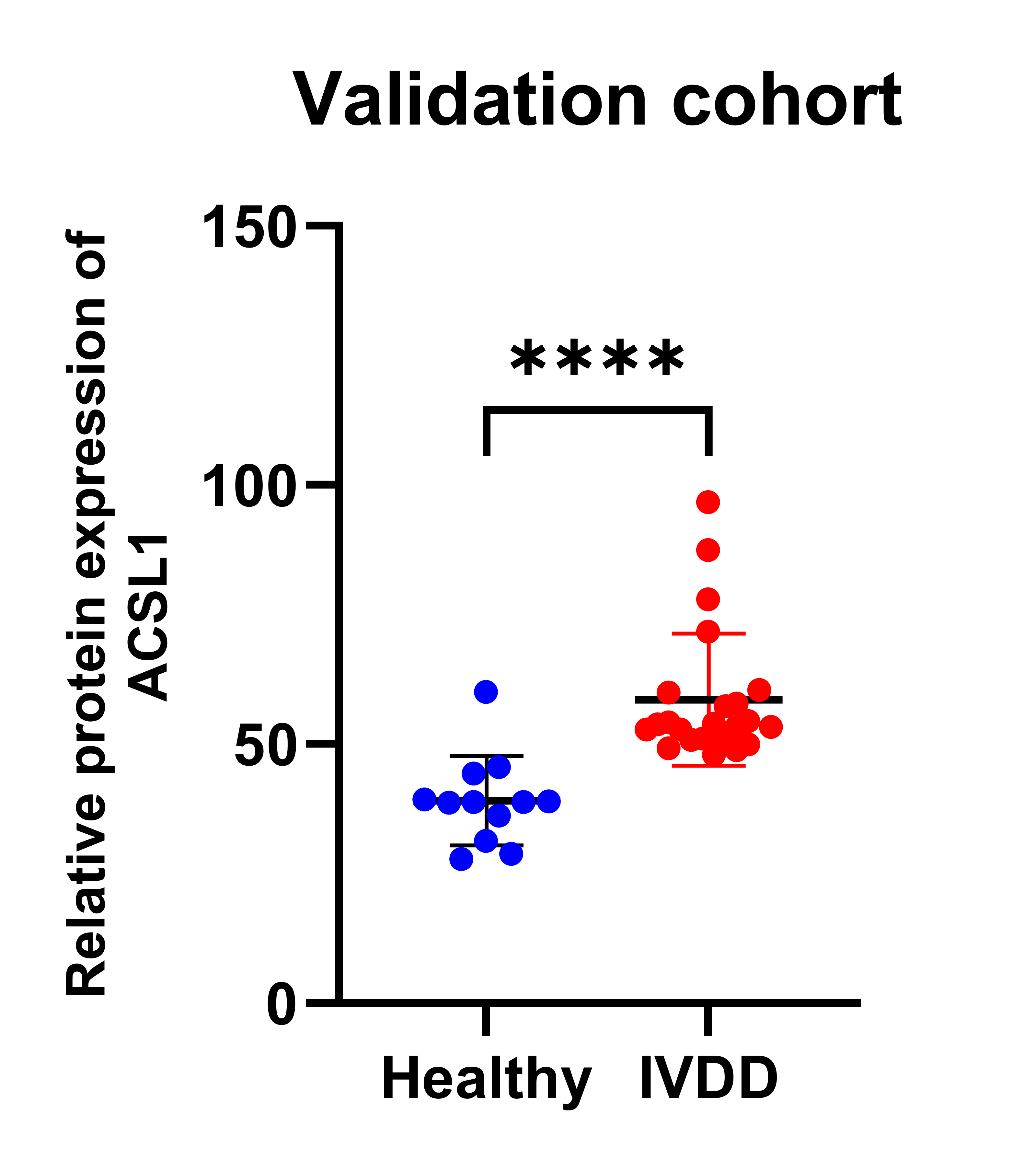

Supplement: Supplementary Figure 1 [file BSR-45-04-BSR20241414-s007.zip › Figure S2.tif]

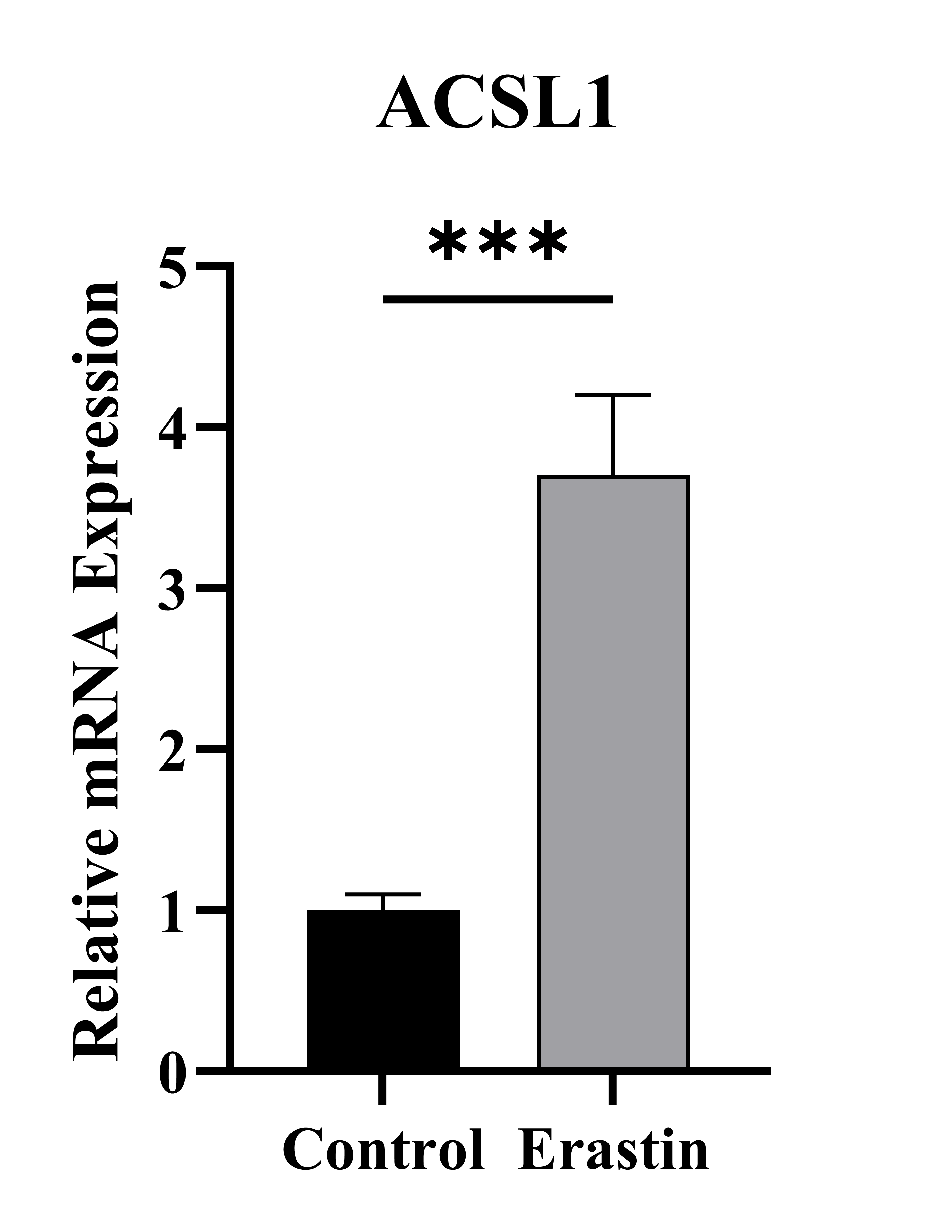

Supplement: Supplementary Figure 2 [file BSR-45-04-BSR20241414-s008.zip › Fig S1/Fig S1.tif]
